# Knowledge of non-healthcare individuals towards cardiopulmonary resuscitation: a cross-sectional study in Riyadh City, Saudi Arabia

**DOI:** 10.1186/s12245-021-00335-y

**Published:** 2021-02-10

**Authors:** Reema M. Alhussein, Mansoor M. Albarrak, Abdulaziz A. Alrabiah, Nawfal A. Aljerian, Hashim M. Bin Salleeh, Ahmad S. Hersi, Tariq A. Wani, Zohair A. Al Aseri

**Affiliations:** 1 College of Medicine, Dar Al Uloom University, Riyadh, Saudi Arabia; 2 Department of Emergency Medicine, College of Medicine, King Saud University, P.O. Box 7805, Riyadh, 11472 Kingdom of Saudi Arabia; 3 Department of Emergency Medicine, King Abdulaziz Medical City, King Saud Bin Abdulaziz University for Health Sciences, Ministry of National Guard, Riyadh, Saudi Arabia; 4 Medical Referrals Center-Ministry of Health, Riyadh, Saudi Arabia; 5 Department of Cardiac Sciences, College of Medicine, King Saud University, P.O. Box 7805, Riyadh, 11472 Kingdom of Saudi Arabia; 6 Research Center, King Fahad Medical City, Riyadh, Saudi Arabia; 7 Departments of Emergency Medicine and Critical Care, College of Medicine, King Saud University, P.O. Box 7805, Riyadh, 11472 Kingdom of Saudi Arabia

**Keywords:** Cardio-pulmonary resuscitation, Basic Cardiac Life Support, CPR, Knowledge, Resuscitation

## Abstract

**Background:**

Most sudden cardiac arrests occur at home, with low rates of bystander cardiopulmonary resuscitation being performed. We aimed to assess knowledge of cardiopulmonary resuscitation among individuals in Riyadh City, Saudi Arabia, who are not involved in health care.

**Methods:**

A community-based cross-sectional study was conducted between January and February 2020 in 4 different areas in Riyadh City: North, South, East, and West. The participants were surveyed using a validated self-administered questionnaire. The Statistical Package for Social Sciences version 25.0 was used for inferential statistics and binary logistic regression analysis.

**Results:**

A total of 856 participants completed the questionnaire, 51.8% were unaware of cardiopulmonary resuscitation. Only 4.4% of the participants had attended a formal cardiopulmonary resuscitation training course, 5.1% were campaign attendees, and 38.7% acquired their experience through the media. Having a higher level of education was positively associated with having knowledge of cardiopulmonary resuscitation. The main concern among attendees of cardiopulmonary resuscitation training courses and campaigns was legal issues, whereas inadequate knowledge was the major barrier for those who had learned about cardiopulmonary resuscitation through the media.

**Conclusion:**

The level of knowledge of cardiopulmonary resuscitation among non-health care individuals in Riyadh City was found to be insufficient. Therefore, coordinated efforts among different authorities should be considered to implement a structured strategy aiming to increase awareness and knowledge of cardiopulmonary resuscitation among non-health care individuals.

## Background

Despite increasing efforts in prevention and treatment, sudden cardiac arrest (SCA) remains a global health issue. However, the incidence of SCA varies according to the region [1]. In the USA, the annual incidence of mortality from out-of-hospital cardiac arrest (OHCA) is estimated to be 90%, causing 356,000 deaths annually [2]. Locally, 74% of SCAs occurred at home with low rates of bystander cardiopulmonary resuscitation (CPR) being performed, and the family members are almost always at the scene before the arrival of emergency medical services (EMS) [3].

The beneficial effects of CPR depend entirely on the knowledge and performance of the bystanders in determining the survival probabilities [4]. Increased public education and improved programme quality and access to public automatic external defibrillators (AEDs) are associated with better outcomes [5–7]

In different community settings, numerous studies have been conducted to assess the CPR-related knowledge and training status of individuals not involved in health care. A Saudi study based in Jeddah found that only 28.7% of respondents stated that they had received CPR training before, compared to 25.6% in China, 29% in Jordan, 40.7% in Izmir and 55.7% in Australia [8–12]. Notably, the highest percentages were reported in countries where there are various efforts to increase CPR training, such as in Canada and Germany, with percentages of 64% and 83.2%, respectively [13, 14]. Furthermore, numerous countries have linked driver license application with mandatory Basic Life Support (BLS) training courses, as in Slovenia and Japan [4, 15]. However, there are a variety of barriers associated with performing CPR; a lack of knowledge and confidence, a fear of harming the victim, and legal consequences make respondents less likely to perform CPR on a stranger [13, 16].With respect to a family member, 94.2% are willing to perform CPR [8].

In Riyadh City, there is a paucity of data regarding knowledge of and attitudes towards CPR, with most of the published studies focusing on different groups of medical and non-medical populations. In the current study, we primarily aimed to assess the CPR-related knowledge of individuals not involved in health care in Riyadh City. Our secondary aim was to assess the willingness of the community to perform CPR and to address the barriers associated with performing CPR.

## Methods

### Study design

A community-based cross-sectional study was conducted over a 2-month period between January and February 2020. The targeted population was individuals not involved in health care who were from different areas of Riyadh City, Saudi Arabia. All individuals were at least 18 years of age; both males and females, those of both Saudi and non-Saudi nationality and those living in Riyadh City were included. Individuals who were younger than 18 years of age or who were studying or graduating from any medical specialty were excluded because their programmes had mandatory BLS courses.

### Sampling and sample size

The study sample size was calculated based on the study conducted in Jeddah, Saudi Arabia [8], which reported that only 32.7% of their respondents knew how to give a chest compressions. With a prevalence of 32.7%, we used a 95% CI with 5% as a margin of error, which gave a minimal sample size of 339. The inter-cluster variance was controlled by multiplying by the design effect, equal to 2.5. Hence, the total number needed for conducting the survey was *n* = 848. An equal proportion of *n* = 212 surveys from each of the distinguished areas were required to ensure that the sample was representative of the total population.

### Data collection tool

Data were collected using a self-administered questionnaire that was adopted from the existing literature addressing the same research question [8, 11]. Some of the questions were rephrased. The modified questionnaire was based on the 2015 American Heart Association guidelines [17]. Content validity analysis of the questionnaire was performed by an expert panel from the fields of emergency medicine and cardiology. The Statistical Package for Social Sciences (SPSS) 25.0 software was used for determining the reliability with Cronbach’s alpha (*r* = 0.70) for the pilot sample. The questionnaire consisted of 26 items. The first section captured the sociodemographic characteristics of the participants (age, gender, residential area, level of education, and occupational status). The other sections assessed their knowledge of CPR, ability to identify the signs and recognition of SCA victims, previous SCA experiences, willingness and barriers to perform CPR, application of CPR and Saudi Red Crescent number. A medical translation specialist translated the questionnaire into Arabic. It was back-translated to English by another medical translation expert to ascertain the accuracy and precision of the translation. Forty such participants who understood both Arabic and English equally responded to both versions during the pilot testing of the questionnaire.

### Data collection

Riyadh City was divided into four different areas (North, South, East, and West) and clustered stratified sampling was chosen. Each area was visited by one dedicated research coordinator who was familiar with the inclusion and exclusion criteria; no intervention occurred during participants’ completion of the questionnaire, and the participants were provided assurance of confidentiality. We recruited participants from shopping centers, universities, social centres, walkaways, parks, gyms, coffee shops, and supermarkets to ensure participant diversity. The study centers were chosen randomly and were similar in the four selected areas, and the participants from each cluster were recruited using convenience sampling.

### Data management and analysis

The study was designed to present all the data in the nominal and ordinal structure. Data validation was performed in Microsoft Excel 16.0, and the clean data were used in the statistical analysis. All the data were described as frequencies and frequency percentages. Participants’ knowledge source was associated with all other study variables pertinent to their knowledge and indicated diverse and non-overlapping responses. All the associations were measured by chi-square analysis, and Fisher’s exact test was applied wherever the expected frequency of a particular cell was below 5. A binary logistic regression was applied to investigate the relationship between CPR knowledge and demographic variables. All inferences were drawn at 95% CI. Statistical analyses were performed with SPSS 25.0 software. *p* values < 0.05 were considered statistically significant.

## Results

The surveyed sample of 856 participants was recruited from 4 different residential areas. The sociodemographic characteristics of the participants are presented in Table 1. A total of 54.2% of the study sample was composed of males, and 54.4% was composed of those aged 25–44 years, which was expected because this age group represents the majority of the population according to the Saudi General Authority for Statistics [18].

**Table 1:** Sociodemographic characteristics of the study participants

Characteristics		*N* = 856	%
Age	18–24	258	30.1
	25–34	297	34.7
	35–44	169	19.7
	45–54	88	10.3
	55–64	44	5.1
Gender	Male	464	54.2
	Female	392	45.8
Area of residence	North Riyadh	212	24.8
	South Riyadh	217	25.3
	East Riyadh	212	24.8
	West Riyadh	215	25.1
Educational level	Elementary school	8	0.9
	Middle school	32	3.7
	High school	379	44.3
	Diploma	57	6.7
	Bachelor degree	332	38.8
	Postgraduate	48	5.6
Occupational status	Student	152	17.8
	Employee	482	56.3
	Self employed	42	4.9
	Housewife	86	10
	Retired	43	5
	Unemployed	51	6

Participants were divided depending on their awareness of CPR; those who were unaware of CPR (51.8%) were precluded from the CPR knowledge questions analysis. The participants with awareness (48.2%) were subdivided into 3 groups as per their reported source of attainment of CPR knowledge: 4.4% of participants received training through attending a formal CPR course, whereas 5.1% attended campaigns run by health care institutions, and 38.7% acquired their experience through different social media networks, medical-related series and movies. The educational backgrounds of formal CPR training course attendees were bachelor’s degree holders (55.3%), high school (18.4%), postgraduate (15.8%), and diploma certified (10.5%). Table 2 shows the knowledge sources of the participants.

**Table 2:** Cardiopulmonary resuscitation knowledge source

Source of knowledge	*N*	(%)
Formal cardiopulmonary resuscitation course	38	4.4
Campaigns presented by health care institutions	44	5.1
Media sources (internet, television)	331	38.7
Unaware of cardiopulmonary resuscitation	443	51.8

Educational level and occupational status depicted in Table 3 show a significant association with knowledge. However, age, gender, and residential area were not significant. With regard to occupational status, housewives were less likely to have a positive attitude towards CPR than were students (OR = 0.23; 95% CI = 0.12–0.46; *p* < 0.001). In addition, postgraduate qualified participants were more likely to have knowledge of CPR than were participants with an elementary-level education (OR = 7.37; 95% CI = 1.29–42.13; *p* = 0.025).

**Table 3:** Binary logistic regression analysis of the factors associated with CPR knowledge

Characteristic	Description	CPR aware	Exp (B)	95% C.I. for EXP(B)		*p* value	Overall *p* value
				Lower	Upper		
Age	18–24	120 (29.2)	1	1	1	0.374	0.784
	25–34	150 (36.5)	1.41	0.92	2.15	0.113	
	35–44	76 (18.5)	1.15	0.7	1.89	0.576	
	45–54	44 (10.7)	1.66	0.91	3.03	0.098	
	55–64	21 (5.1)	1.28	0.46	3.59	0.637	
Gender	Male	226 (55.0)	1	1	1	1	0.659
	Female	185 (45.0)	1.09	0.79	1.49	0.602	
Residential area	North	111 (27.0)	1	1	1	0.316	0.132
	South	107 (26.0)	1.42	0.93	2.18	0.106	
	East	104 (25.3)	1.12	0.74	1.69	0.587	
	West	89 (21.7)	1.02	0.66	1.58	0.92	
Level of education	Elementary school	2 (.5)	1	1	1	< 0.001	< 0.001
	Middle school	8 (1.9)	0.92	0.15	5.65	0.924	
	High school	149 (36.3)	1.62	0.31	8.42	0.568	
	Diploma	29 (7.1)	3.08	0.56	16.99	0.198	
	Bachelor degree	189 (46.0)	3.94	0.76	20.41	0.102	
	Postgraduate	34 (8.3)	7.37	1.29	42.13	0.025	
Occupational status	Student	89 (21.7)	1	1	1	< 0.001	0.008
	Employee	224 (54.5)	0.37	0.23	0.59	< 0.001	
	Self employed	24 (5.8)	0.53	0.23	1.19	0.123	
	Housewife	29 (7.1)	0.23	0.12	0.46	< 0.001	
	Retired	20 (4.9)	0.37	0.13	1.05	0.062	
	Unemployed	25 (6.1)	0.39	0.2	0.78	0.008	
Constant			0.58			0.54	

*CI* confidence interval, *CPR* cardiopulmonary resuscitation

Of all those surveyed, 51.8% listed the absence of a pulse. The absence of breathing was identified by 48.1% and a loss of consciousness by 44.9%. Six percent of the participants failed to identify any of the listed signs of SCA. In terms of recognition of SCA victims, the three different aware groups were compared with respect to the evaluation of consciousness and breathing. Among all of the participants with CPR awareness (*n* = 413), 27.6% and 29% could correctly assess consciousness and breathing, respectively. The evaluation rate of consciousness and breathing was found to be higher among those who received training through campaigns (31.8% and 43.2%), respectively, with statistical significance in terms of breathing evaluation (*p* = 0.037).

When all participants were asked about the correct response in approaching a SCA victim, it was observed that those who attended a formal CPR training course had the highest percentage of reporting the need to check consciousness and breathing, start CPR, and call EMS, followed by other groups (*p* < 0.001). The majority of unaware participants (54%) stated that they would not intervene. Table 4 summarizes the participants’ approach in the case of SCA.

**Table 4:** Association between approaching a victim in SCA and CPR knowledge source among all participants

Variables	Source of knowledge				
	Formal CPR course *n* (%)	Campaign *n* (%)	Median (%)	Unaware of CPR *n* (%)	*p* value
Checking consciousness level	22 (57.9)	22 (50.0)	27 (8.2)	37 (8.4)	< 0.001
Checking breathing	35 (92.1)	38 (86.4)	134 (40.5)	95 (21.4)	< 0.001
Starting a CPR	29 (76.3)	31 (70.5)	34 (10.3)	0 (0.0)	< 0.001
Calling EMS	28 (73.7)	28 (63.6)	190 (57.4)	185 (41.8)	< 0.001
Watching only	0 (0.0)	0 (0.0)	113 (34.1)	239 (54)	< 0.001

*CPR* cardiopulmonary resuscitation, *SCA* sudden cardiac arrest, *EMS* emergency medical services

Table 5 reveals a statistically significant difference in terms of applying CPR among those who had received CPR training (*p* < 0.001); 50% of those who attended a formal CPR course knew how to apply all 3 CPR procedures. Regarding the application of CPR in terms of the proper rate of compression, the ratio, the location of compression, and the force that must be applied during compression, those who attended a formal CPR course showed a higher rate of correct answers than did those in the other groups (*p* < 0.001).

**Table 5:** Association between practical application of CPR and knowledge source among aware participants

Variables	Source of knowledge			
	Formal CPR course *n* (%)	Campaign *n* (%)	Media *n* (%)	*p* value
Procedures				
Opening an airway	21 (55.3)	21 (47.7)	32 (9.7)	< 0.001
Give mouth-mouth ventilation	25 (65.8)	24 (54.5)	28 (8.5)	< 0.001
Starting chest compression	36 (94.7)	40 (90.9)	48 (14.5)	< 0.001
Giving mouth to mouth-start chest compression	24 (63.2)	22 (50.0)	19 (5.7)	< 0.001
Those knowing three procedures	19 (50.0)	16 (36.4)	9 (2.7)	< 0.001
I do not know	0 (0.0)	0 (0.0)	257 (77.6)	< 0.001
Which one of the following choices is the correct location for chest compressions in CPR?				
Upper part of the chest	7 (18.4)	6 (13.6)	37 (11.2)	< 0.001
Middle part of the chest	19 (50.0)	28 (63.6)	93 (28.1)	
Lower part of the chest ^†^	12 (31.6)	10 (22.7)	31 (9.4)	
I do not know	0 (0.0)	0 (0.0)	170 (51.4)	
What is the ratio of chest compressions to mouth-to-mouth ventilation in CPR?				
5:1	11 (29)	18 (40.9)	9 (2.7)	< 0.001
15:2	16 (42)	15 (34.1)	6 (1.8)	
30:2 ^†^	11 (29)	11 (25)	7 (2.1)	
I do not know	0 (0.0%)	0 (0.0%)	309 (93.4)	
What is the rate of chest compressions in CPR?				
150 per min	4 (10.5)	3 (6.8)	5 (1.5)	< 0.001
100–120 per min ^†^	11 (28.9)	12 (27.3)	12 (3.6)	
50 per min	23 (60.5)	29 (65.9)	17 (5.1)	
I do not know	0 (0.0)	0 (0.0)	297 (89.7)	
What is the length of strength that should be given during chest compressions in CPR?				
A depth of 1–2 cm	12 (31.6)	21 (47.7)	9 (2.7)	< 0.001
A depth of 5–6 cm ^†^	21 (55.3)	22 (50.0)	24 (7.3)	
A depth of 6–10 cm	5 (13.2)	1 (2.3)	4 (1.2)	
I do not know	0 (0.0)	0 (0.0)	294 (88.8)	

*CPR* cardiopulmonary resuscitation

†Correct answer

In the case of SCA, the results revealed that among all of our aware participants, 36% were willing to perform CPR for their family members (*p* < 0.001), followed by 24.2% who were willing to perform CPR for a friend, 21.5% for a neighbour, and 15.7% for a stranger. However, 4.6% stated that they would not intervene in the case of SCA. Nonetheless, the majority of participants who gained their experience through media sources stated that they do not feel confident in performing CPR. Legal issues were the most common barrier preventing those who received hands-on training from performing CPR on a stranger. Breaking a rib bone was the most common concern regarding the application of CPR on a family member or a friend (*p* = 0.002). Inadequate knowledge and skills related to CPR remain the main barriers that prevent participants who gained their experience through media from performing CPR (*p* < 0.001). Further details for willingness and barriers to perform CPR are presented in Table 6.

**Table 6:** Association between willingness and barriers of performing CPR and CPR knowledge source among aware participants

Variables	Source of knowledge			
	Formal CPR course *n* (%)	Campaign *n* (%)	Media *n* (%)	*p* value
In case of SCA, who would you perform CPR for?				
A family member	30 (78.9)	34 (77.3)	85 (25.7)	< 0.001
A friend	25 (65.8)	26 (59.1)	49 (14.8)	< 0.001
A neighbour	23 (60.5)	24 (54.5)	42 (12.7)	< 0.001
A stranger	20 (52.6)	22 (50.0)	23 (6.9)	< 0.001
I know how to perform CPR, but will not do it	7 (18.4)	7 (15.9)	5 (1.5)	< 0.001
I do not feel confident to perform CPR	0 (0.0)	0 (0.0)	274 (82.8)	< 0.001
What barriers may stop you from performing CPR for a family member or a friend?				
Breaking a rib bone	16 (42.1)	18 (40.9)	74 (22.4)	0.002
Causing an internal organ damage	10 (26.3)	14 (31.8)	45 (13.6)	0.002
Legal issues	4 (10.5)	4 (9.1)	16 (4.8)	0.154
Stopping heart from work	5 (13.2)	8 (18.2)	26 (7.9)	<0.001
Infection	4 (10.5)	3 (6.8)	5 (1.5)	0.003
I have no fear, I will perform CPR	18 (47.4)	16 (36.4)	14 (4.2)	<0.001
Inadequate knowledge and skill of CPR	0 (0.0)	1 (2.3)	277 (83.7)	< 0.001
What barriers may stop you from performing CPR for a stranger?				
Breaking a rib bone	8 (21.1)	13(29.5)	68 (20.5)	0.393
Causing an internal organ damage	9 (23.7)	15 (34.1)	41 (12.4)	< 0.001
Stopping heart from work	7 (18.4)	11 (25.0)	18 (5.4)	< 0.001
Legal issues	16 (42.1)	15 (34.1)	72 (21.8)	0.008
Infection	4 (10.5)	5 (11.4)	15 (4.5)	0.054
I have no fear, I will perform CPR	14 (36.8)	15 (34.1)	9 (2.7)	< 0.001
Inadequate knowledge and skill of CPR	0 (0.0)	1 (2.3)	275 (83.1)	< 0.001

*CPR* cardiopulmonary resuscitation, *SCA* sudden cardiac arrest

Among all of the participant, only 45.2% of all participants were aware of the current Saudi Red Crescent number (997), as the results revealed a significant association between positive CPR awareness and recognizing the Saudi Red Crescent number (*p* < 0.001). There was an association between having attended a campaign and selecting the correct Saudi Red Crescent number, as 77.3% of them selected it correctly.

## Discussion

The initiation of CPR before the arrival of professional help doubles the 30-day survival rate, which indicates the need for community involvement in understanding the practical application of CPR to provide resuscitation effectively and safely [19, 20]. We aimed to assess the CPR-related knowledge of individuals not involved in health care and their willingness to perform it and to uncover the barriers preventing them from performing CPR. Compared to other studies on this topic, our study is unique, as it sub-grouped the aware participants and compared them depending on the sources from which they received their knowledge. Other studies were limited to specific regions, conducted their surveys by email or social media or included health care individuals.

In our study, we found a poor level of awareness of CPR based on participant reports; 51.8% had no awareness of CPR. In comparison, only 10% of Australian and Chinese populations had never heard of CPR [9, 12]. However, the other half of our participants (48.2%) acquired their knowledge through different sources. Based on the subgroup analysis, 4.4% received their training through a formal CPR course, 5.1% through campaigns, and 38.7% through the media. The training rate in Riyadh City seemed higher than that in Hong Kong (21%), Jeddah (28.7%), Jordan (29%), and Izmir (40.3%) [8, 10, 11, 21]. In contrast, our rate of training was lower than that of countries where CPR courses are a mandatory requirement. In Slovenia, 69.4% of the respondents had received previous CPR training, which was associated with mandatory training during the acquisition of driver’s licenses [4]. In Denmark, multinational initiatives were taken to increase the rate of bystander CPR, and after implementing different strategies, the rate of bystander CPR increased significantly from 21.1% in 2001 to 44.9% in 2010 [22]. High percentages of trained individuals were also reported in Scotland (52%) [23], Australia (56%) [12], the UK (59%) [24], Canada (64%) [13], and the USA (65%) [25].

Participants showed some logical awareness of the signs of SCA. A high proportion of the participants stated that the absence of a pulse, breathing, and the loss of consciousness were the most common signs of SCA (51.8%, 48.1%, and 44.9%, respectively). Our findings were consistent with those of Özbilgin et al., as 60.7% selected the absence of a pulse, 49.3% selected the absence of breathing, and 40.7% selected the loss of consciousness [11]. On the other hand, our results were higher than those reported in Jeddah, as 24.7%, 36.8%, and 40.7% of their respondents could properly identify signs of SCA, absence of pulse, breathing, and loss of consciousness, respectively [8]. When participants were asked about their recognition of an SCA victim, among all of the aware participants, 27.6% and 29% could correctly assess consciousness and breathing, respectively. Overall, our findings were higher than those of Jeddah City, where among those who received training, only 12.8% and 20.2% could correctly assess consciousness and breathing, respectively [8]. Similarly, poor results were reported in Izmir and Jordan as well [10, 11]. However, those who acquired their training through campaigns had a higher set of skills in recognizing an unconscious victim (31.8%) and identifying the correct way to assess breathing (43.2%). The results may reflect the quality of campaigns and the importance of refresher training courses for those who had previously attended a CPR course.

The participants were asked how to approach someone with SCA; the majority of participants (76.3%) who had taken a formal CPR course and 70.5% who were campaign attendees, respectively; (*p* < 0.001) stated that they would start CPR. In contrast, 34.1% of the participants who gained their CPR experience through the media and 54% of those who were totally unaware of CPR stated that they would only watch without intervening (*p* < 0.001). In our study, 14.3% of all participants had encountered a situation that required CPR, with most of those victims being a family member. Among those, only 6.5% started CPR. The results indicate the importance of training programmes that encourage individuals not involved in health care to perform CPR in a real-life situation when it is indicated. Among Turkish participants, 18.6% stated that they had previously witnessed SCA, and only 3.6% performed CPR [11]. A lower rate of CPR was also reported in Jordan and Jeddah [8, 10]

With regard to the practical application of CPR according to the American Heart Association guidelines, self-reported answers revealed poor knowledge among aware participants regarding the application of CPR. Among all aware participants, only 15.7% stated that they would perform both chest compression and mouth-to-mouth breathing. However, 10.6% stated that they would perform all 3 procedures. Overall, only 12.8% knew the correct location of compression, and 7% knew the ratio of compressions to ventilation. The percentages of correct responses for rate and depth were 8.4% and 16.2%, respectively. Our participants had the same level of poor knowledge shown in previous studies [10, 11]. However, the rates of correct answers were found to be significantly higher among the formal CPR course attendees group (*p* < 0.001). Our findings revealed discrepancies among those who gained their experience through the media. This is justified by their different perceptions of having sufficient knowledge based on what they demonstrated from the media. In addition, these devastating results reflect the quality of the theoretical details given in different sources and the necessity of regular high-quality CPR training and refresher courses.

In the case of SCA, numerous studies reported that high percentages of their respondents would begin CPR for their family members [8, 10, 11]. Among all our aware participants, 36% showed no hesitation to perform CPR for their family members, followed by for a friend, neighbor, and finally a stranger. The main reason for a lack of willingness to conduct CPR among those who gained their experience through the media was being unconfident in performing CPR on any of the aforementioned individuals (*p* < 0.001), reflecting the significance of hands-on training in the other groups. However, 4.6% stated that they knew how to perform CPR but would not perform it (*p* < 0.001). There are certain barriers that may affect the willingness of the aware participants to begin CPR. With regard to family members, 11.6% stated that nothing would prevent them from performing CPR (*p* < 0.001), whereas 26.1% had a fear of breaking a rib bone (*p* = 0.002). The most common reason for not conducting CPR on a stranger among formal CPR course attendees and campaign attendees was legal issues 42.1% and 34.1%, respectively. This raises our appeal to local authorities to create a law to encourage the public to step in when indicated, such as the ‘good Samaritan law’, which is enacted in a number of countries, such as the USA and Korea. The law is enacted to protect individuals from any consequences and offer legal protection when they assist in good faith and deliver medical assistance for strangers in emergencies [26, 27].

A total of 54.8% of all participants were unaware of the current emergency contact number (Saudi Red Crescent number 997). Among the participants, 77.3% of campaign attendees selected the correct answer, 57.9% of formal CPR course attendees, 52.6% of media-obtained participants, and 35.4% of those unaware of CPR. The results revealed that the campaign attendees group were the most aware of the correct number, which might reflect that local campaigns focus on giving the correct Saudi Red Crescent number, while CPR course materials are developed abroad, where the universal emergency service number (911) differs from that used in Saudi Arabia.

This study has some limitations. The status of knowledge of CPR and training skills was based on participants’ self-report. In addition, CPR training skills were only tested theoretically, not practically, as the latter was not feasible for our study participants. Lastly, we did not ask whether the formal CPR course attendees training was recent or not.

## Conclusion and recommendations

Our study highlighted the limited knowledge of CPR among individuals in Riyadh City who were not involved in health care. This may have an impact on individuals’ ability to act in the case of SCA. Therefore, coordinated efforts among different authorities should be considered to implement a structured strategy aiming to increase awareness and knowledge of CPR. Accessible high-quality CPR courses, incorporate CPR material into educational curricula, in addition to regular hands-on training and refresher courses, could address this issue. Finally, since the media is considered a major source of information for the majority of our aware participants, greater focus on expanding the educational material such as “How to apply hands-on CPR” and guidance to authorized CPR centers near them, and to raise awareness by directed campaigns such as “ Why should you learn how to perform CPR” in addition to monitoring and approval of CPR educational content is needed by experts in the field.

## Data Availability

The data used to support the findings of this study are available from the corresponding author upon request.

## Abbreviations

SCA: Sudden cardiac arrest
OHCA: Out of hospital cardiac arrest
CPR: Cardiopulmonary resuscitation
EMS: Emergency medical services
AED: Automated external defibrillator
BLS: Basic Life Support
CI: Confidence interval
SPSS: Statistical Package for Social Sciences
